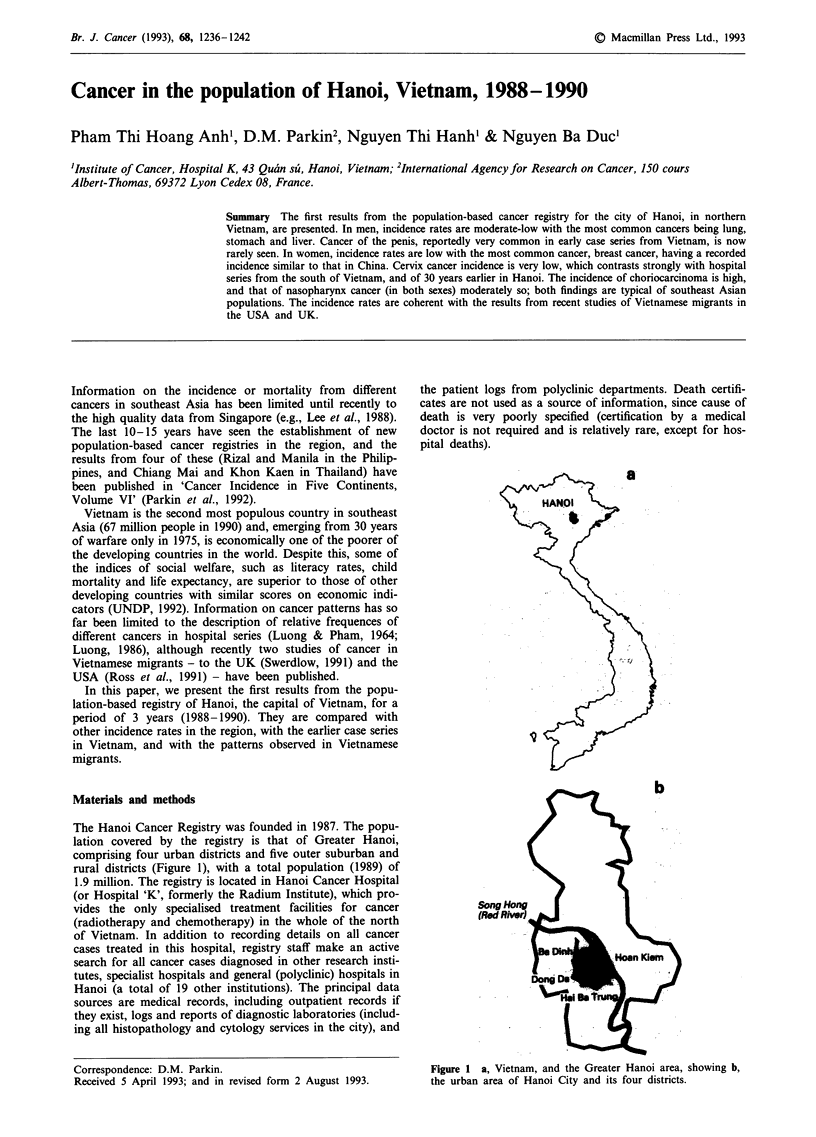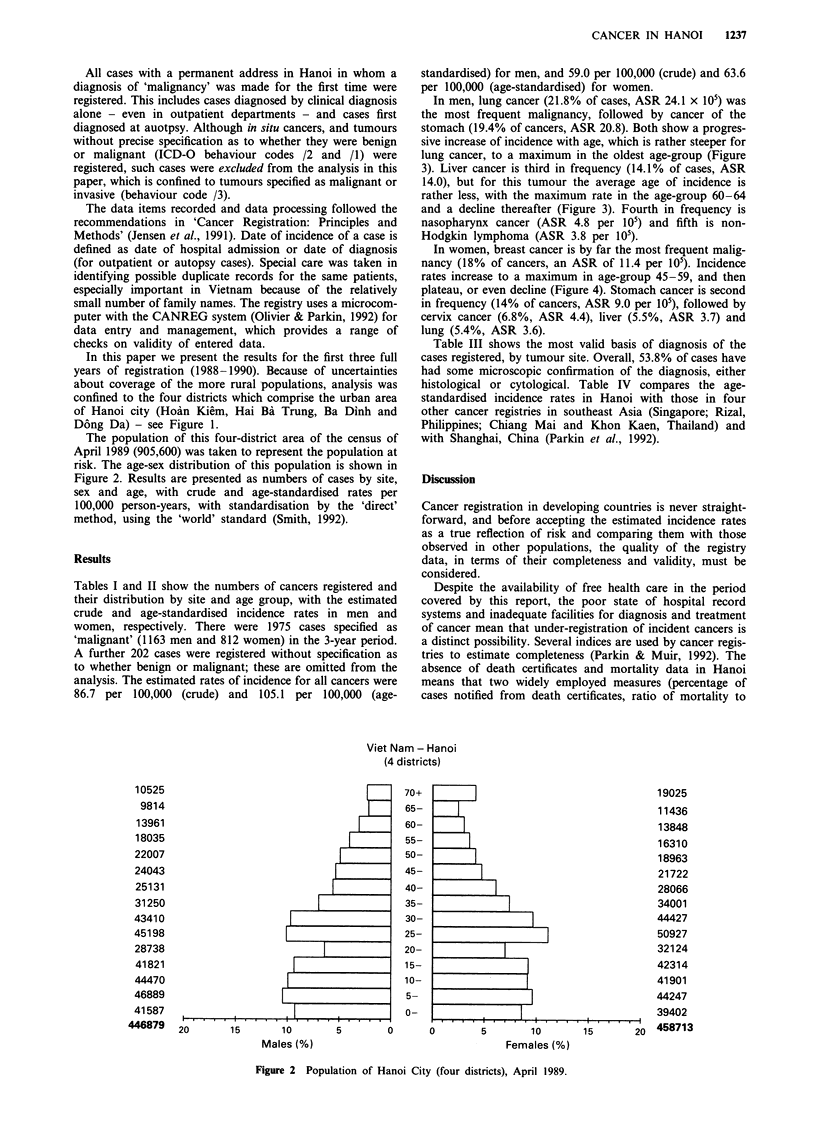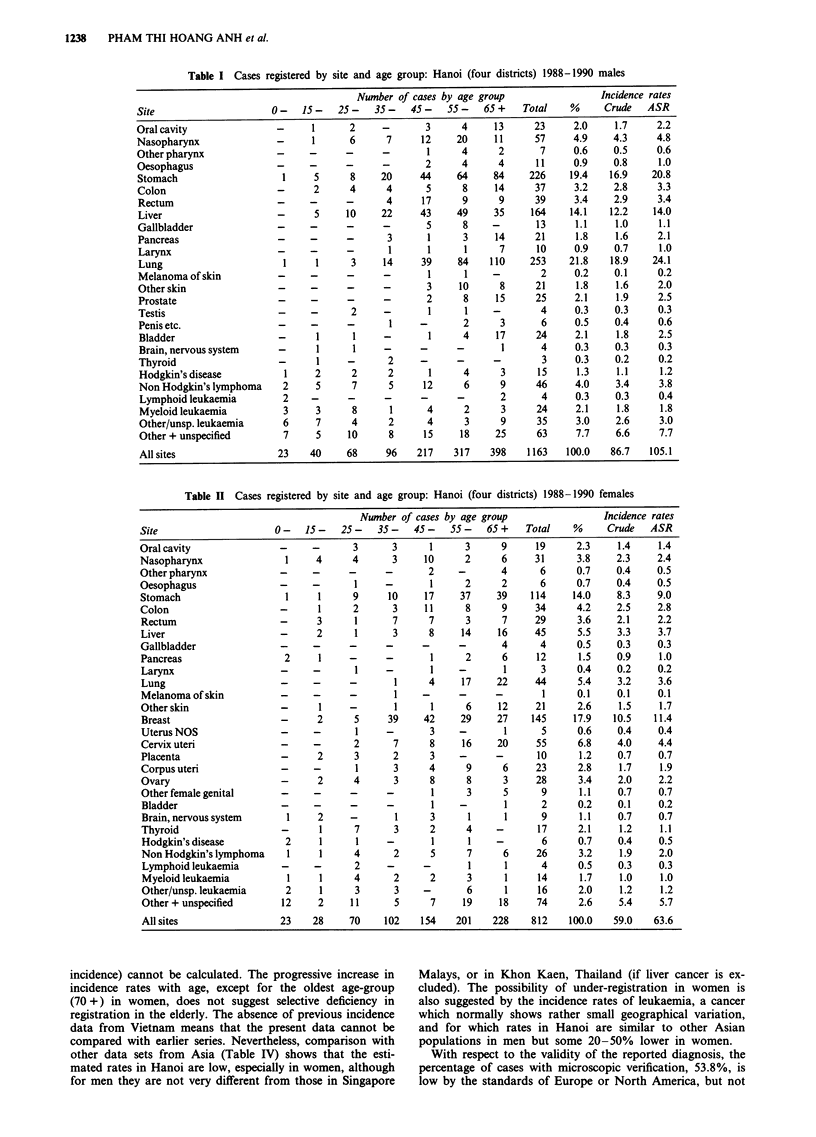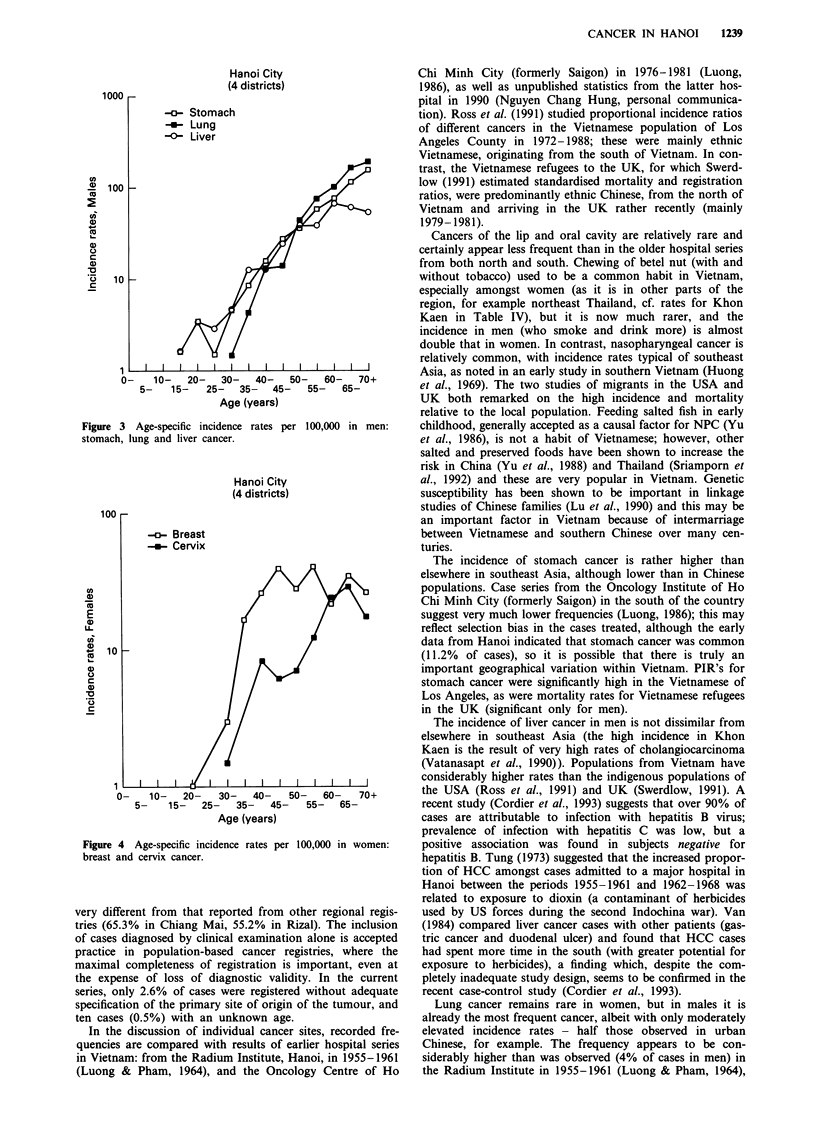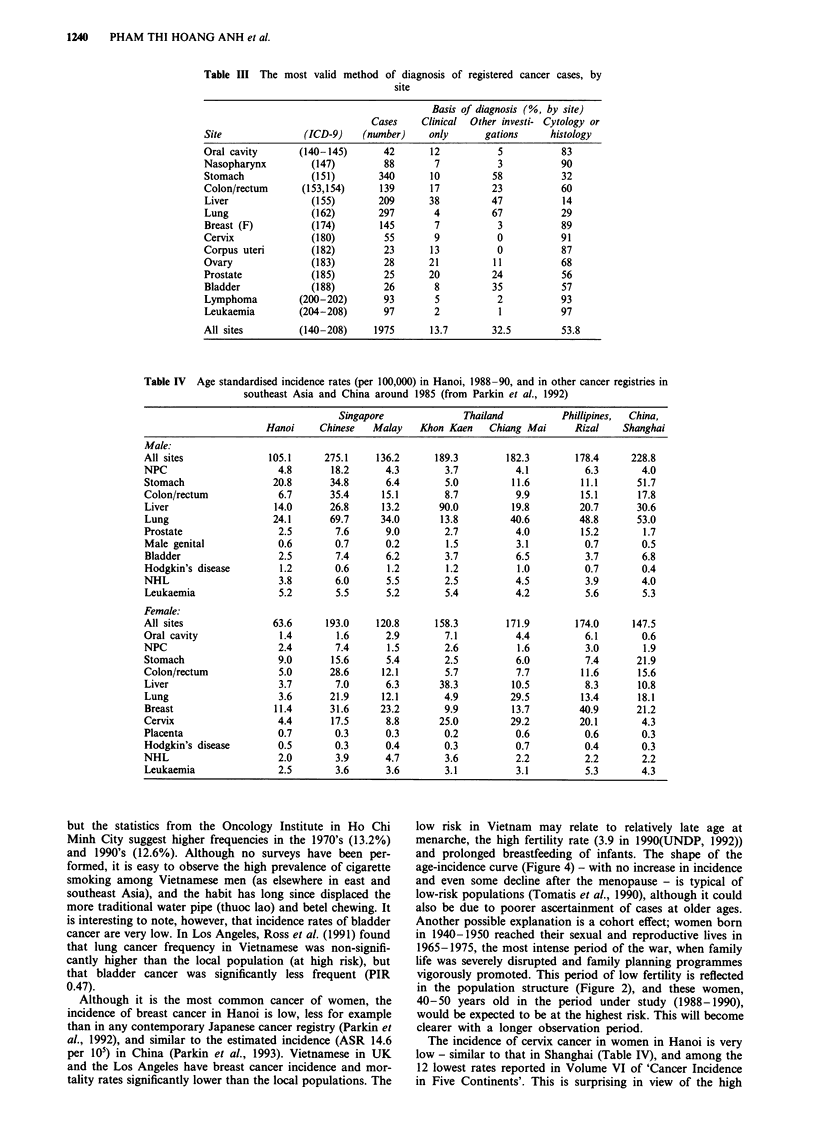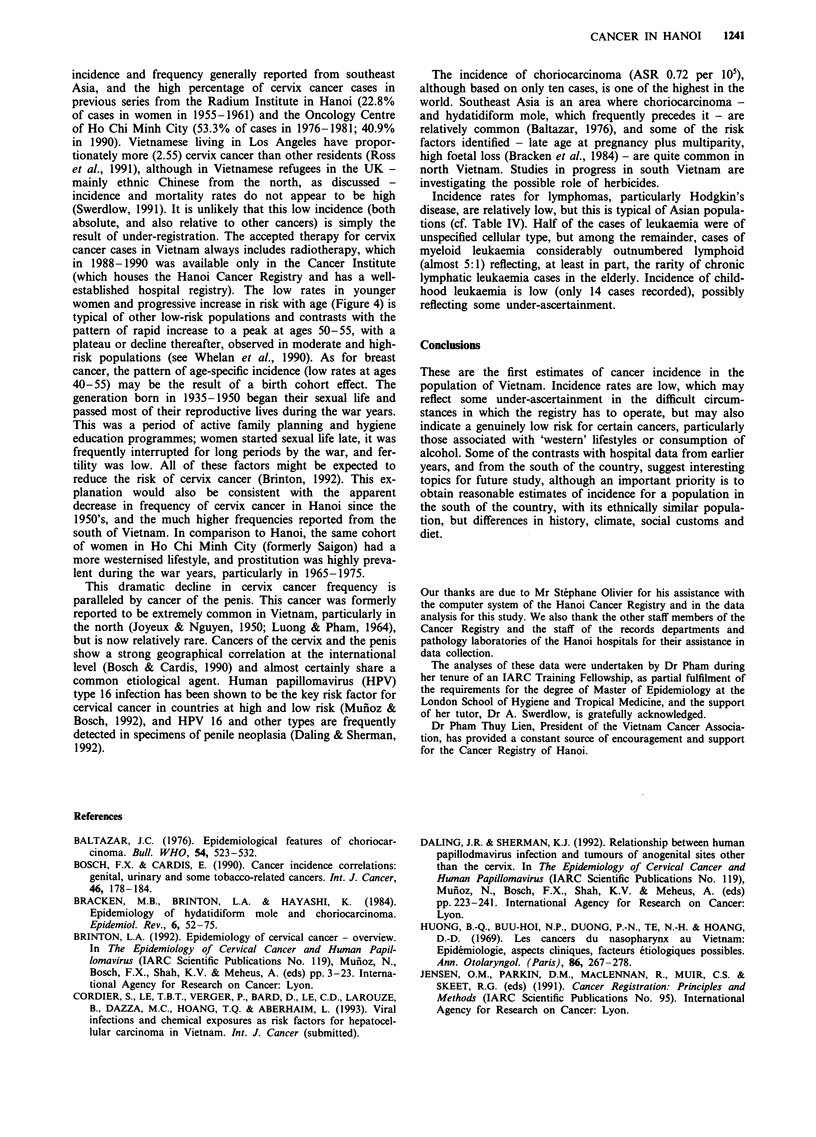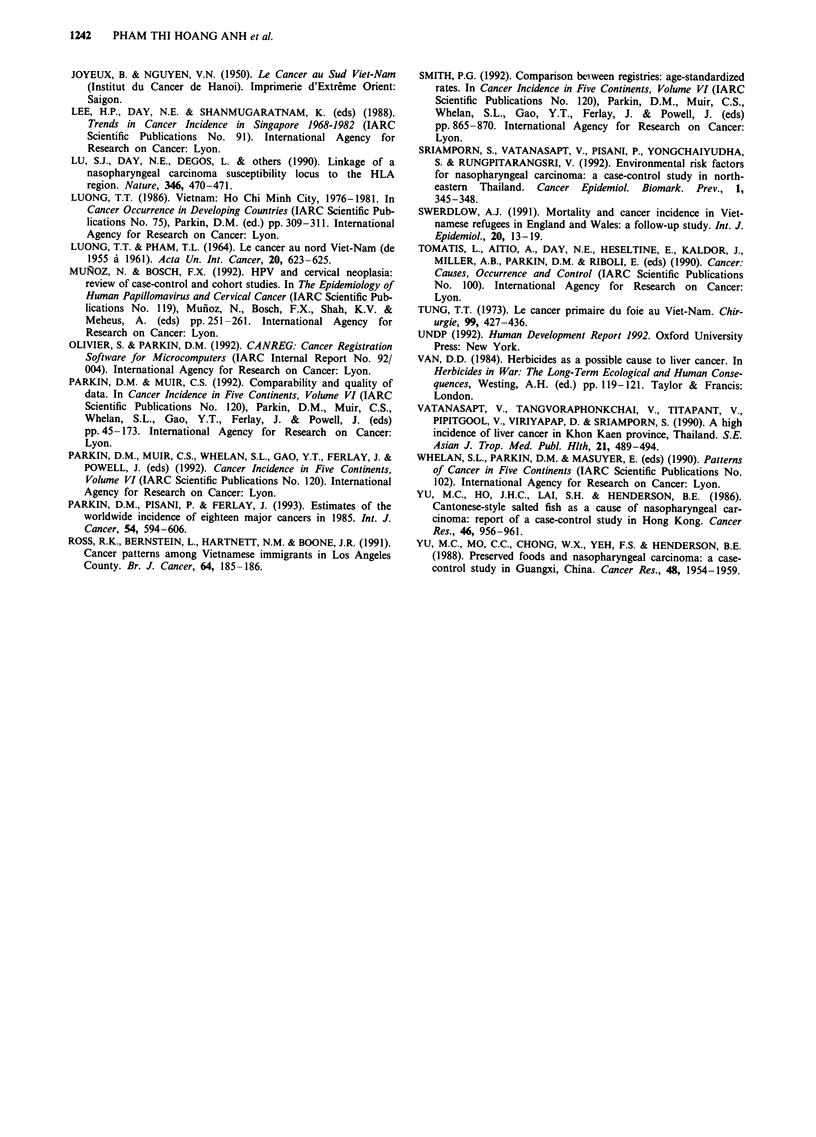# Cancer in the population of Hanoi, Vietnam, 1988-1990.

**DOI:** 10.1038/bjc.1993.511

**Published:** 1993-12

**Authors:** P. T. Anh, D. M. Parkin, N. T. Hanh, N. B. Duc

**Affiliations:** Institute of Cancer, Hospital K, Hanoi, Vietnam.

## Abstract

The first results from the population-based cancer registry for the city of Hanoi, in northern Vietnam, are presented. In men, incidence rates are moderate-low with the most common cancers being lung, stomach and liver. Cancer of the penis, reportedly very common in early case series from Vietnam, is now rarely seen. In women, incidence rates are low with the most common cancer, breast cancer, having a recorded incidence similar to that in China. Cervix cancer incidence is very low, which contrasts strongly with hospital series from the south of Vietnam, and of 30 years earlier in Hanoi. The incidence of choriocarcinoma is high, and that of nasopharynx cancer (in both sexes) moderately so; both findings are typical of southeast Asian populations. The incidence rates are coherent with the results from recent studies of Vietnamese migrants in the USA and UK.


Br. J. Cancer (1993), 68, 1236-1242 © Macmillan Press Ltd., 1993

### Cancer in the population of Hanoi, Vietnam, 1988-1990

Pham Thi Hoang Anh[1], D.M. Parkin[2], Nguyen Thi Hanh[1] & Nguyen Ba Duc[1]

[1]*Institute of Cancer, Hospital K, 43 Quán sú, Hanoi, Vietnam;* [2]*International Agency for Research on Cancer, 150 cours Albert-Thomas, 69372 Lyon Cedex 08, France.*

**Summary** The first results from the population-based cancer registry for the city of Hanoi, in northern Vietnam, are presented. In men, incidence rates are moderate-low with the most common cancers being lung, stomach and liver. Cancer of the penis, reportedly very common in early case series from Vietnam, is now rarely seen. In women, incidence rates are low with the most common cancer, breast cancer, having a recorded incidence similar to that in China. Cervix cancer incidence is very low, which contrasts strongly with hospital series from the south of Vietnam, and of 30 years earlier in Hanoi. The incidence of choriocarcinoma is high, and that of nasopharynx cancer (in both sexes) moderately so; both findings are typical of southeast Asian populations. The incidence rates are coherent with the results from recent studies of Vietnamese migrants in the USA and UK.

Information on the incidence or mortality from different cancers in southeast Asia has been limited until recently to the high quality data from Singapore (e.g., Lee *et al.*, 1988). The last 10-15 years have seen the establishment of new population-based cancer registries in the region, and the results from four of these (Rizal and Manila in the Philippines, and Chiang Mai and Khon Kaen in Thailand) have been published in 'Cancer Incidence in Five Continents, Volume VI' (Parkin *et al.*, 1992).

Vietnam is the second most populous country in southeast Asia (67 million people in 1990) and, emerging from 30 years of warfare only in 1975, is economically one of the poorer of the developing countries in the world. Despite this, some of the indices of social welfare, such as literacy rates, child mortality and life expectancy, are superior to those of other developing countries with similar scores on economic indicators (UNDP, 1992). Information on cancer patterns has so far been limited to the description of relative frequences of different cancers in hospital series (Luong & Pham, 1964; Luong, 1986), although recently two studies of cancer in Vietnamese migrants - to the UK (Swerdlow, 1991) and the USA (Ross *et al.*, 1991) - have been published.

In this paper, we present the first results from the population-based registry of Hanoi, the capital of Vietnam, for a period of 3 years (1988-1990). They are compared with other incidence rates in the region, with the earlier case series in Vietnam, and with the patterns observed in Vietnamese migrants.

### Materials and methods

The Hanoi Cancer Registry was founded in 1987. The population covered by the registry is that of Greater Hanoi, comprising four urban districts and five outer suburban and rural districts (Figure 1), with a total population (1989) of 1.9 million. The registry is located in Hanoi Cancer Hospital (or Hospital 'K', formerly the Radium Institute), which provides the only specialised treatment facilities for cancer (radiotherapy and chemotherapy) in the whole of the north of Vietnam. In addition to recording details on all cancer cases treated in this hospital, registry staff make an active search for all cancer cases diagnosed in other research institutes, specialist hospitals and general (polyclinic) hospitals in Hanoi (a total of 19 other institutions). The principal data sources are medical records, including outpatient records if they exist, logs and reports of diagnostic laboratories (including all histopathology and cytology services in the city), and the patient logs from polyclinic departments. Death certificates are not used as a source of information, since cause of death is very poorly specified (certification by a medical doctor is not required and is relatively rare, except for hospital deaths).

**Figure 1** **a**, Vietnam, and the Greater Hanoi area, showing **b**, the urban area of Hanoi City and its four districts.

Correspondence: D.M. Parkin.
Received 5 April 1993; and in revised form 2 August 1993.

All cases with a permanent address in Hanoi in whom a diagnosis of 'malignancy' was made for the first time were registered. This includes cases diagnosed by clinical diagnosis alone – even in outpatient departments – and cases first diagnosed at auotpsy. Although *in situ* cancers, and tumours without precise specification as to whether they were benign or malignant (ICD-O behaviour codes /2 and /1) were registered, such cases were *excluded* from the analysis in this paper, which is confined to tumours specified as malignant or invasive (behaviour code /3).

The data items recorded and data processing followed the recommendations in 'Cancer Registration: Principles and Methods' (Jensen *et al.*, 1991). Date of incidence of a case is defined as date of hospital admission or date of diagnosis (for outpatient or autopsy cases). Special care was taken in identifying possible duplicate records for the same patients, especially important in Vietnam because of the relatively small number of family names. The registry uses a microcomputer with the CANREG system (Olivier & Parkin, 1992) for data entry and management, which provides a range of checks on validity of entered data.

In this paper we present the results for the first three full years of registration (1988–1990). Because of uncertainties about coverage of the more rural populations, analysis was confined to the four districts which comprise the urban area of Hanoi city (Hoàn Kiêm, Hai Bà Trung, Ba Dình and Dông Da) – see Figure 1.

The population of this four-district area of the census of April 1989 (905,600) was taken to represent the population at risk. The age-sex distribution of this population is shown in Figure 2. Results are presented as numbers of cases by site, sex and age, with crude and age-standardised rates per 100,000 person-years, with standardisation by the 'direct' method, using the 'world' standard (Smith, 1992).

### Results

Tables I and II show the numbers of cancers registered and their distribution by site and age group, with the estimated crude and age-standardised incidence rates in men and women, respectively. There were 1975 cases specified as 'malignant' (1163 men and 812 women) in the 3-year period. A further 202 cases were registered without specification as to whether benign or malignant; these are omitted from the analysis. The estimated rates of incidence for all cancers were 86.7 per 100,000 (crude) and 105.1 per 100,000 (age-standardised) for men, and 59.0 per 100,000 (crude) and 63.6 per 100,000 (age-standardised) for women.

In men, lung cancer (21.8% of cases, ASR 24.1 × $10^5$) was the most frequent malignancy, followed by cancer of the stomach (19.4% of cancers, ASR 20.8). Both show a progressive increase of incidence with age, which is rather steeper for lung cancer, to a maximum in the oldest age-group (Figure 3). Liver cancer is third in frequency (14.1% of cases, ASR 14.0), but for this tumour the average age of incidence is rather less, with the maximum rate in the age-group 60–64 and a decline thereafter (Figure 3). Fourth in frequency is nasopharynx cancer (ASR 4.8 per $10^5$) and fifth is non-Hodgkin lymphoma (ASR 3.8 per $10^5$).

In women, breast cancer is by far the most frequent malignancy (18% of cancers, an ASR of 11.4 per $10^5$). Incidence rates increase to a maximum in age-group 45–59, and then plateau, or even decline (Figure 4). Stomach cancer is second in frequency (14% of cancers, ASR 9.0 per $10^5$), followed by cervix cancer (6.8%, ASR 4.4), liver (5.5%, ASR 3.7) and lung (5.4%, ASR 3.6).

Table III shows the most valid basis of diagnosis of the cases registered, by tumour site. Overall, 53.8% of cases have had some microscopic confirmation of the diagnosis, either histological or cytological. Table IV compares the age-standardised incidence rates in Hanoi with those in four other cancer registries in southeast Asia (Singapore; Rizal, Philippines; Chiang Mai and Khon Kaen, Thailand) and with Shanghai, China (Parkin *et al.*, 1992).

### Discussion

Cancer registration in developing countries is never straightforward, and before accepting the estimated incidence rates as a true reflection of risk and comparing them with those observed in other populations, the quality of the registry data, in terms of their completeness and validity, must be considered.

Despite the availability of free health care in the period covered by this report, the poor state of hospital record systems and inadequate facilities for diagnosis and treatment of cancer mean that under-registration of incident cancers is a distinct possibility. Several indices are used by cancer registries to estimate completeness (Parkin & Muir, 1992). The absence of death certificates and mortality data in Hanoi means that two widely employed measures (percentage of cases notified from death certificates, ratio of mortality to

**Figure 2** Population of Hanoi City (four districts), April 1989.

**Table I** Cases registered by site and age group: Hanoi (four districts) 1988–1990 males

| Site | *Number of cases by age group* 0– | 15– | 25– | 35– | 45– | 55– | 65+ | *Total* | *%* | *Incidence rates* Crude | ASR |
|---|---|---|---|---|---|---|---|---|---|---|---|
| Oral cavity | – | 1 | 2 | – | 3 | 4 | 13 | 23 | 2.0 | 1.7 | 2.2 |
| Nasopharynx | – | 1 | 6 | 7 | 12 | 20 | 11 | 57 | 4.9 | 4.3 | 4.8 |
| Other pharynx | – | – | – | – | 1 | 4 | 2 | 7 | 0.6 | 0.5 | 0.6 |
| Oesophagus | – | – | – | – | 2 | 4 | 4 | 11 | 0.9 | 0.8 | 1.0 |
| Stomach | 1 | 5 | 8 | 20 | 44 | 64 | 84 | 226 | 19.4 | 16.9 | 20.8 |
| Colon | – | 2 | 4 | 4 | 5 | 8 | 14 | 37 | 3.2 | 2.8 | 3.3 |
| Rectum | – | – | – | 4 | 17 | 9 | 9 | 39 | 3.4 | 2.9 | 3.4 |
| Liver | – | 5 | 10 | 22 | 43 | 49 | 35 | 164 | 14.1 | 12.2 | 14.0 |
| Gallbladder | – | – | – | – | 5 | 8 | – | 13 | 1.1 | 1.0 | 1.1 |
| Pancreas | – | – | – | 3 | 1 | 3 | 14 | 21 | 1.8 | 1.6 | 2.1 |
| Larynx | – | – | – | 1 | 1 | 1 | 7 | 10 | 0.9 | 0.7 | 1.0 |
| Lung | 1 | 1 | 3 | 14 | 39 | 84 | 110 | 253 | 21.8 | 18.9 | 24.1 |
| Melanoma of skin | – | – | – | – | 1 | 1 | – | 2 | 0.2 | 0.1 | 0.2 |
| Other skin | – | – | – | – | 3 | 10 | 8 | 21 | 1.8 | 1.6 | 2.0 |
| Prostate | – | – | – | – | 2 | 8 | 15 | 25 | 2.1 | 1.9 | 2.5 |
| Testis | – | – | 2 | – | 1 | 1 | – | 4 | 0.3 | 0.3 | 0.3 |
| Penis etc. | – | – | – | 1 | – | 2 | 3 | 6 | 0.5 | 0.4 | 0.6 |
| Bladder | – | 1 | 1 | – | 1 | 4 | 17 | 24 | 2.1 | 1.8 | 2.5 |
| Brain, nervous system | – | 1 | 1 | – | – | – | 1 | 4 | 0.3 | 0.3 | 0.3 |
| Thyroid | – | 1 | – | 2 | – | – | – | 3 | 0.3 | 0.2 | 0.2 |
| Hodgkin's disease | 1 | 2 | 2 | 2 | 1 | 4 | 3 | 15 | 1.3 | 1.1 | 1.2 |
| Non Hodgkin's lymphoma | 2 | 5 | 7 | 5 | 12 | 6 | 9 | 46 | 4.0 | 3.4 | 3.8 |
| Lymphoid leukaemia | 2 | – | – | – | – | – | 2 | 4 | 0.3 | 0.3 | 0.4 |
| Myeloid leukaemia | 3 | 3 | 8 | 1 | 4 | 2 | 3 | 24 | 2.1 | 1.8 | 1.8 |
| Other/unsp. leukaemia | 6 | 7 | 4 | 2 | 4 | 3 | 9 | 35 | 3.0 | 2.6 | 3.0 |
| Other + unspecified | 7 | 5 | 10 | 8 | 15 | 18 | 25 | 63 | 7.7 | 6.6 | 7.7 |
| All sites | 23 | 40 | 68 | 96 | 217 | 317 | 398 | 1163 | 100.0 | 86.7 | 105.1 |

**Table II** Cases registered by site and age group: Hanoi (four districts) 1988–1990 females

| Site | *Number of cases by age group* 0– | 15– | 25– | 35– | 45– | 55– | 65+ | *Total* | *%* | *Incidence rates* Crude | ASR |
|---|---|---|---|---|---|---|---|---|---|---|---|
| Oral cavity | – | – | 3 | 3 | 1 | 3 | 9 | 19 | 2.3 | 1.4 | 1.4 |
| Nasopharynx | 1 | 4 | 4 | 3 | 10 | 2 | 6 | 31 | 3.8 | 2.3 | 2.4 |
| Other pharynx | – | – | – | – | 2 | – | 4 | 6 | 0.7 | 0.4 | 0.5 |
| Oesophagus | – | – | 1 | – | 1 | 2 | 2 | 6 | 0.7 | 0.4 | 0.5 |
| Stomach | 1 | 1 | 9 | 10 | 17 | 37 | 39 | 114 | 14.0 | 8.3 | 9.0 |
| Colon | – | 1 | 2 | 3 | 11 | 8 | 9 | 34 | 4.2 | 2.5 | 2.8 |
| Rectum | – | 3 | 1 | 7 | 7 | 3 | 7 | 29 | 3.6 | 2.1 | 2.2 |
| Liver | – | 2 | 1 | 3 | 8 | 14 | 16 | 45 | 5.5 | 3.3 | 3.7 |
| Gallbladder | – | – | – | – | – | – | 4 | 4 | 0.5 | 0.3 | 0.3 |
| Pancreas | 2 | 1 | – | – | 1 | 2 | 6 | 12 | 1.5 | 0.9 | 1.0 |
| Larynx | – | – | 1 | – | 1 | – | 1 | 3 | 0.4 | 0.2 | 0.2 |
| Lung | – | – | – | 1 | 4 | 17 | 22 | 44 | 5.4 | 3.2 | 3.6 |
| Melanoma of skin | – | – | – | 1 | – | – | – | 1 | 0.1 | 0.1 | 0.1 |
| Other skin | – | 1 | – | 1 | 1 | 6 | 12 | 21 | 2.6 | 1.5 | 1.7 |
| Breast | – | 2 | 5 | 39 | 42 | 29 | 27 | 145 | 17.9 | 10.5 | 11.4 |
| Uterus NOS | – | – | 1 | – | 3 | – | 1 | 5 | 0.6 | 0.4 | 0.4 |
| Cervix uteri | – | – | 2 | 7 | 8 | 16 | 20 | 55 | 6.8 | 4.0 | 4.4 |
| Placenta | – | 2 | 3 | 2 | 3 | – | – | 10 | 1.2 | 0.7 | 0.7 |
| Corpus uteri | – | – | 1 | 3 | 4 | 9 | 6 | 23 | 2.8 | 1.7 | 1.9 |
| Ovary | – | 2 | 4 | 3 | 8 | 8 | 3 | 28 | 3.4 | 2.0 | 2.2 |
| Other female genital | – | – | – | – | 1 | 3 | 5 | 9 | 1.1 | 0.7 | 0.7 |
| Bladder | – | – | – | – | 1 | – | 1 | 2 | 0.2 | 0.1 | 0.2 |
| Brain, nervous system | 1 | 2 | – | 1 | 3 | 1 | 1 | 9 | 1.1 | 0.7 | 0.7 |
| Thyroid | – | 1 | 7 | 3 | 2 | 4 | – | 17 | 2.1 | 1.2 | 1.1 |
| Hodgkin's disease | 2 | 1 | 1 | – | 1 | 1 | – | 6 | 0.7 | 0.4 | 0.5 |
| Non Hodgkin's lymphoma | 1 | 1 | 4 | 2 | 5 | 7 | 6 | 26 | 3.2 | 1.9 | 2.0 |
| Lymphoid leukaemia | – | – | 2 | – | – | 1 | 1 | 4 | 0.5 | 0.3 | 0.3 |
| Myeloid leukaemia | 1 | 1 | 4 | 2 | 2 | 3 | 1 | 14 | 1.7 | 1.0 | 1.0 |
| Other/unsp. leukaemia | 2 | 1 | 3 | 3 | – | 6 | 1 | 16 | 2.0 | 1.2 | 1.2 |
| Other + unspecified | 12 | 2 | 11 | 5 | 7 | 19 | 18 | 74 | 2.6 | 5.4 | 5.7 |
| All sites | 23 | 28 | 70 | 102 | 154 | 201 | 228 | 812 | 100.0 | 59.0 | 63.6 |

incidence) cannot be calculated. The progressive increase in incidence rates with age, except for the oldest age-group (70+) in women, does not suggest selective deficiency in registration in the elderly. The absence of previous incidence data from Vietnam means that the present data cannot be compared with earlier series. Nevertheless, comparison with other data sets from Asia (Table IV) shows that the estimated rates in Hanoi are low, especially in women, although for men they are not very different from those in Singapore Malays, or in Khon Kaen, Thailand (if liver cancer is excluded). The possibility of under-registration in women is also suggested by the incidence rates of leukaemia, a cancer which normally shows rather small geographical variation, and for which rates in Hanoi are similar to other Asian populations in men but some 20–50% lower in women.

With respect to the validity of the reported diagnosis, the percentage of cases with microscopic verification, 53.8%, is low by the standards of Europe or North America, but not

**Figure 3** Age-specific incidence rates per 100,000 in men: stomach, lung and liver cancer.

**Figure 4** Age-specific incidence rates per 100,000 in women: breast and cervix cancer.

very different from that reported from other regional registries (65.3% in Chiang Mai, 55.2% in Rizal). The inclusion of cases diagnosed by clinical examination alone is accepted practice in population-based cancer registries, where the maximal completeness of registration is important, even at the expense of loss of diagnostic validity. In the current series, only 2.6% of cases were registered without adequate specification of the primary site of origin of the tumour, and ten cases (0.5%) with an unknown age.

In the discussion of individual cancer sites, recorded frequencies are compared with results of earlier hospital series in Vietnam: from the Radium Institute, Hanoi, in 1955–1961 (Luong & Pham, 1964), and the Oncology Centre of Ho Chi Minh City (formerly Saigon) in 1976–1981 (Luong, 1986), as well as unpublished statistics from the latter hospital in 1990 (Nguyen Chang Hung, personal communication). Ross *et al.* (1991) studied proportional incidence ratios of different cancers in the Vietnamese population of Los Angeles County in 1972–1988; these were mainly ethnic Vietnamese, originating from the south of Vietnam. In contrast, the Vietnamese refugees to the UK, for which Swerdlow (1991) estimated standardised mortality and registration ratios, were predominantly ethnic Chinese, from the north of Vietnam and arriving in the UK rather recently (mainly 1979–1981).

Cancers of the lip and oral cavity are relatively rare and certainly appear less frequent than in the older hospital series from both north and south. Chewing of betel nut (with and without tobacco) used to be a common habit in Vietnam, especially amongst women (as it is in other parts of the region, for example northeast Thailand, cf. rates for Khon Kaen in Table IV), but it is now much rarer, and the incidence in men (who smoke and drink more) is almost double that in women. In contrast, nasopharyngeal cancer is relatively common, with incidence rates typical of southeast Asia, as noted in an early study in southern Vietnam (Huong *et al.*, 1969). The two studies of migrants in the USA and UK both remarked on the high incidence and mortality relative to the local population. Feeding salted fish in early childhood, generally accepted as a causal factor for NPC (Yu *et al.*, 1986), is not a habit of Vietnamese; however, other salted and preserved foods have been shown to increase the risk in China (Yu *et al.*, 1988) and Thailand (Sriamporn *et al.*, 1992) and these are very popular in Vietnam. Genetic susceptibility has been shown to be important in linkage studies of Chinese families (Lu *et al.*, 1990) and this may be an important factor in Vietnam because of intermarriage between Vietnamese and southern Chinese over many centuries.

The incidence of stomach cancer is rather higher than elsewhere in southeast Asia, although lower than in Chinese populations. Case series from the Oncology Institute of Ho Chi Minh City (formerly Saigon) in the south of the country suggest very much lower frequencies (Luong, 1986); this may reflect selection bias in the cases treated, although the early data from Hanoi indicated that stomach cancer was common (11.2% of cases), so it is possible that there is truly an important geographical variation within Vietnam. PIR's for stomach cancer were significantly high in the Vietnamese of Los Angeles, as were mortality rates for Vietnamese refugees in the UK (significant only for men).

The incidence of liver cancer in men is not dissimilar from elsewhere in southeast Asia (the high incidence in Khon Kaen is the result of very high rates of cholangiocarcinoma (Vatanasapt *et al.*, 1990)). Populations from Vietnam have considerably higher rates than the indigenous populations of the USA (Ross *et al.*, 1991) and UK (Swerdlow, 1991). A recent study (Cordier *et al.*, 1993) suggests that over 90% of cases are attributable to infection with hepatitis B virus; prevalence of infection with hepatitis C was low, but a positive association was found in subjects *negative* for hepatitis B. Tung (1973) suggested that the increased proportion of HCC amongst cases admitted to a major hospital in Hanoi between the periods 1955–1961 and 1962–1968 was related to exposure to dioxin (a contaminant of herbicides used by US forces during the second Indochina war). Van (1984) compared liver cancer cases with other patients (gastric cancer and duodenal ulcer) and found that HCC cases had spent more time in the south (with greater potential for exposure to herbicides), a finding which, despite the completely inadequate study design, seems to be confirmed in the recent case-control study (Cordier *et al.*, 1993).

Lung cancer remains rare in women, but in males it is already the most frequent cancer, albeit with only moderately elevated incidence rates – half those observed in urban Chinese, for example. The frequency appears to be considerably higher than was observed (4% of cases in men) in the Radium Institute in 1955–1961 (Luong & Pham, 1964),

**Table III** The most valid method of diagnosis of registered cancer cases, by site

| Site | (ICD-9) | Cases (number) | Basis of diagnosis (%, by site) Clinical only | Other investi- gations | Cytology or histology |
|---|---|---|---|---|---|
| Oral cavity | (140–145) | 42 | 12 | 5 | 83 |
| Nasopharynx | (147) | 88 | 7 | 3 | 90 |
| Stomach | (151) | 340 | 10 | 58 | 32 |
| Colon/rectum | (153,154) | 139 | 17 | 23 | 60 |
| Liver | (155) | 209 | 38 | 47 | 14 |
| Lung | (162) | 297 | 4 | 67 | 29 |
| Breast (F) | (174) | 145 | 7 | 3 | 89 |
| Cervix | (180) | 55 | 9 | 0 | 91 |
| Corpus uteri | (182) | 23 | 13 | 0 | 87 |
| Ovary | (183) | 28 | 21 | 11 | 68 |
| Prostate | (185) | 25 | 20 | 24 | 56 |
| Bladder | (188) | 26 | 8 | 35 | 57 |
| Lymphoma | (200–202) | 93 | 5 | 2 | 93 |
| Leukaemia | (204–208) | 97 | 2 | 1 | 97 |
| All sites | (140–208) | 1975 | 13.7 | 32.5 | 53.8 |

**Table IV** Age standardised incidence rates (per 100,000) in Hanoi, 1988–90, and in other cancer registries in southeast Asia and China around 1985 (from Parkin *et al.*, 1992)

| | Hanoi | Singapore Chinese | Singapore Malay | Thailand Khon Kaen | Thailand Chiang Mai | Phillipines, Rizal | China, Shanghai |
|---|---|---|---|---|---|---|---|
| *Male:* | | | | | | | |
| All sites | 105.1 | 275.1 | 136.2 | 189.3 | 182.3 | 178.4 | 228.8 |
| NPC | 4.8 | 18.2 | 4.3 | 3.7 | 4.1 | 6.3 | 4.0 |
| Stomach | 20.8 | 34.8 | 6.4 | 5.0 | 11.6 | 11.1 | 51.7 |
| Colon/rectum | 6.7 | 35.4 | 15.1 | 8.7 | 9.9 | 15.1 | 17.8 |
| Liver | 14.0 | 26.8 | 13.2 | 90.0 | 19.8 | 20.7 | 30.6 |
| Lung | 24.1 | 69.7 | 34.0 | 13.8 | 40.6 | 48.8 | 53.0 |
| Prostate | 2.5 | 7.6 | 9.0 | 2.7 | 4.0 | 15.2 | 1.7 |
| Male genital | 0.6 | 0.7 | 0.2 | 1.5 | 3.1 | 0.7 | 0.5 |
| Bladder | 2.5 | 7.4 | 6.2 | 3.7 | 6.5 | 3.7 | 6.8 |
| Hodgkin's disease | 1.2 | 0.6 | 1.2 | 1.2 | 1.0 | 0.7 | 0.4 |
| NHL | 3.8 | 6.0 | 5.5 | 2.5 | 4.5 | 3.9 | 4.0 |
| Leukaemia | 5.2 | 5.5 | 5.2 | 5.4 | 4.2 | 5.6 | 5.3 |
| *Female:* | | | | | | | |
| All sites | 63.6 | 193.0 | 120.8 | 158.3 | 171.9 | 174.0 | 147.5 |
| Oral cavity | 1.4 | 1.6 | 2.9 | 7.1 | 4.4 | 6.1 | 0.6 |
| NPC | 2.4 | 7.4 | 1.5 | 2.6 | 1.6 | 3.0 | 1.9 |
| Stomach | 9.0 | 15.6 | 5.4 | 2.5 | 6.0 | 7.4 | 21.9 |
| Colon/rectum | 5.0 | 28.6 | 12.1 | 5.7 | 7.7 | 11.6 | 15.6 |
| Liver | 3.7 | 7.0 | 6.3 | 38.3 | 10.5 | 8.3 | 10.8 |
| Lung | 3.6 | 21.9 | 12.1 | 4.9 | 29.5 | 13.4 | 18.1 |
| Breast | 11.4 | 31.6 | 23.2 | 9.9 | 13.7 | 40.9 | 21.2 |
| Cervix | 4.4 | 17.5 | 8.8 | 25.0 | 29.2 | 20.1 | 4.3 |
| Placenta | 0.7 | 0.3 | 0.3 | 0.2 | 0.6 | 0.6 | 0.3 |
| Hodgkin's disease | 0.5 | 0.3 | 0.4 | 0.3 | 0.7 | 0.4 | 0.3 |
| NHL | 2.0 | 3.9 | 4.7 | 3.6 | 2.2 | 2.2 | 2.2 |
| Leukaemia | 2.5 | 3.6 | 3.6 | 3.1 | 3.1 | 5.3 | 4.3 |

but the statistics from the Oncology Institute in Ho Chi Minh City suggest higher frequencies in the 1970's (13.2%) and 1990's (12.6%). Although no surveys have been performed, it is easy to observe the high prevalence of cigarette smoking among Vietnamese men (as elsewhere in east and southeast Asia), and the habit has long since displaced the more traditional water pipe (thuoc lao) and betel chewing. It is interesting to note, however, that incidence rates of bladder cancer are very low. In Los Angeles, Ross *et al.* (1991) found that lung cancer frequency in Vietnamese was non-significantly higher than the local population (at high risk), but that bladder cancer was significantly less frequent (PIR 0.47).

Although it is the most common cancer of women, the incidence of breast cancer in Hanoi is low, less for example than in any contemporary Japanese cancer registry (Parkin *et al.*, 1992), and similar to the estimated incidence (ASR 14.6 per $10^5$) in China (Parkin *et al.*, 1993). Vietnamese in UK and the Los Angeles have breast cancer incidence and mortality rates significantly lower than the local populations. The low risk in Vietnam may relate to relatively late age at menarche, the high fertility rate (3.9 in 1990(UNDP, 1992)) and prolonged breastfeeding of infants. The shape of the age-incidence curve (Figure 4) – with no increase in incidence and even some decline after the menopause – is typical of low-risk populations (Tomatis *et al.*, 1990), although it could also be due to poorer ascertainment of cases at older ages. Another possible explanation is a cohort effect; women born in 1940–1950 reached their sexual and reproductive lives in 1965–1975, the most intense period of the war, when family life was severely disrupted and family planning programmes vigorously promoted. This period of low fertility is reflected in the population structure (Figure 2), and these women, 40–50 years old in the period under study (1988–1990), would be expected to be at the highest risk. This will become clearer with a longer observation period.

The incidence of cervix cancer in women in Hanoi is very low – similar to that in Shanghai (Table IV), and among the 12 lowest rates reported in Volume VI of 'Cancer Incidence in Five Continents'. This is surprising in view of the high incidence and frequency generally reported from southeast Asia, and the high percentage of cervix cancer cases in previous series from the Radium Institute in Hanoi (22.8% of cases in women in 1955–1961) and the Oncology Centre of Ho Chi Minh City (53.3% of cases in 1976–1981; 40.9% in 1990). Vietnamese living in Los Angeles have proportionately more (2.55) cervix cancer than other residents (Ross *et al.*, 1991), although in Vietnamese refugees in the UK – mainly ethnic Chinese from the north, as discussed – incidence and mortality rates do not appear to be high (Swerdlow, 1991). It is unlikely that this low incidence (both absolute, and also relative to other cancers) is simply the result of under-registration. The accepted therapy for cervix cancer cases in Vietnam always includes radiotherapy, which in 1988–1990 was available only in the Cancer Institute (which houses the Hanoi Cancer Registry and has a well-established hospital registry). The low rates in younger women and progressive increase in risk with age (Figure 4) is typical of other low-risk populations and contrasts with the pattern of rapid increase to a peak at ages 50–55, with a plateau or decline thereafter, observed in moderate and high-risk populations (see Whelan *et al.*, 1990). As for breast cancer, the pattern of age-specific incidence (low rates at ages 40–55) may be the result of a birth cohort effect. The generation born in 1935–1950 began their sexual life and passed most of their reproductive lives during the war years. This was a period of active family planning and hygiene education programmes; women started sexual life late, it was frequently interrupted for long periods by the war, and fertility was low. All of these factors might be expected to reduce the risk of cervix cancer (Brinton, 1992). This explanation would also be consistent with the apparent decrease in frequency of cervix cancer in Hanoi since the 1950's, and the much higher frequencies reported from the south of Vietnam. In comparison to Hanoi, the same cohort of women in Ho Chi Minh City (formerly Saigon) had a more westernised lifestyle, and prostitution was highly prevalent during the war years, particularly in 1965–1975.

This dramatic decline in cervix cancer frequency is paralleled by cancer of the penis. This cancer was formerly reported to be extremely common in Vietnam, particularly in the north (Joyeux & Nguyen, 1950; Luong & Pham, 1964), but is now relatively rare. Cancers of the cervix and the penis show a strong geographical correlation at the international level (Bosch & Cardis, 1990) and almost certainly share a common etiological agent. Human papillomavirus (HPV) type 16 infection has been shown to be the key risk factor for cervical cancer in countries at high and low risk (Muñoz & Bosch, 1992), and HPV 16 and other types are frequently detected in specimens of penile neoplasia (Daling & Sherman, 1992).

The incidence of choriocarcinoma (ASR 0.72 per $10^5$), although based on only ten cases, is one of the highest in the world. Southeast Asia is an area where choriocarcinoma – and hydatidiform mole, which frequently precedes it – are relatively common (Baltazar, 1976), and some of the risk factors identified – late age at pregnancy plus multiparity, high foetal loss (Bracken *et al.*, 1984) – are quite common in north Vietnam. Studies in progress in south Vietnam are investigating the possible role of herbicides.

Incidence rates for lymphomas, particularly Hodgkin's disease, are relatively low, but this is typical of Asian populations (cf. Table IV). Half of the cases of leukaemia were of unspecified cellular type, but among the remainder, cases of myeloid leukaemia considerably outnumbered lymphoid (almost 5:1) reflecting, at least in part, the rarity of chronic lymphatic leukaemia cases in the elderly. Incidence of childhood leukaemia is low (only 14 cases recorded), possibly reflecting some under-ascertainment.

### Conclusions

These are the first estimates of cancer incidence in the population of Vietnam. Incidence rates are low, which may reflect some under-ascertainment in the difficult circumstances in which the registry has to operate, but may also indicate a genuinely low risk for certain cancers, particularly those associated with 'western' lifestyles or consumption of alcohol. Some of the contrasts with hospital data from earlier years, and from the south of the country, suggest interesting topics for future study, although an important priority is to obtain reasonable estimates of incidence for a population in the south of the country, with its ethnically similar population, but differences in history, climate, social customs and diet.

Our thanks are due to Mr Stéphane Olivier for his assistance with the computer system of the Hanoi Cancer Registry and in the data analysis for this study. We also thank the other staff members of the Cancer Registry and the staff of the records departments and pathology laboratories of the Hanoi hospitals for their assistance in data collection.

The analyses of these data were undertaken by Dr Pham during her tenure of an IARC Training Fellowship, as partial fulfilment of the requirements for the degree of Master of Epidemiology at the London School of Hygiene and Tropical Medicine, and the support of her tutor, Dr A. Swerdlow, is gratefully acknowledged.

Dr Pham Thuy Lien, President of the Vietnam Cancer Association, has provided a constant source of encouragement and support for the Cancer Registry of Hanoi.

### References

BALTAZAR, J.C. (1976). Epidemiological features of choriocarcinoma. *Bull. WHO*, **54**, 523–532.

BOSCH, F.X. & CARDIS, E. (1990). Cancer incidence correlations: genital, urinary and some tobacco-related cancers. *Int. J. Cancer*, **46**, 178–184.

BRACKEN, M.B., BRINTON, L.A. & HAYASHI, K. (1984). Epidemiology of hydatidiform mole and choriocarcinoma. *Epidemiol. Rev.*, **6**, 52–75.

BRINTON, L.A. (1992). Epidemiology of cervical cancer – overview. In *The Epidemiology of Cervical Cancer and Human Papillomavirus* (IARC Scientific Publications No. 119), Muñoz, N., Bosch, F.X., Shah, K.V. & Meheus, A. (eds) pp. 3–23. International Agency for Research on Cancer: Lyon.

CORDIER, S., LE, T.B.T., VERGER, P., BARD, D., LE, C.D., LAROUZE, B., DAZZA, M.C., HOANG, T.Q. & ABERHAIM, L. (1993). Viral infections and chemical exposures as risk factors for hepatocellular carcinoma in Vietnam. *Int. J. Cancer* (submitted).

DALING, J.R. & SHERMAN, K.J. (1992). Relationship between human papillodmavirus infection and tumours of anogenital sites other than the cervix. In *The Epidemiology of Cervical Cancer and Human Papillomavirus* (IARC Scientific Publications No. 119), Muñoz, N., Bosch, F.X., Shah, K.V. & Meheus, A. (eds) pp. 223–241. International Agency for Research on Cancer: Lyon.

HUONG, B.-Q., BUU-HOI, N.P., DUONG, P.-N., TE, N.-H. & HOANG, D.-D. (1969). Les cancers du nasopharynx au Vietnam: Epidémiologie, aspects cliniques, facteurs étiologiques possibles. *Ann. Otolaryngol. (Paris)*, **86**, 267–278.

JENSEN, O.M., PARKIN, D.M., MACLENNAN, R., MUIR, C.S. & SKEET, R.G. (eds) (1991). *Cancer Registration: Principles and Methods* (IARC Scientific Publications No. 95). International Agency for Research on Cancer: Lyon.

JOYEUX, B. & NGUYEN, V.N. (1950). *Le Cancer au Sud Viet-Nam* (Institut du Cancer de Hanoi). Imprimerie d'Extrême Orient: Saigon.

LEE, H.P., DAY, N.E. & SHANMUGARATNAM, K. (eds) (1988). *Trends in Cancer Incidence in Singapore 1968-1982* (IARC Scientific Publications No. 91). International Agency for Research on Cancer: Lyon.

LU, S.J., DAY, N.E., DEGOS, L. & others (1990). Linkage of a nasopharyngeal carcinoma susceptibility locus to the HLA region. *Nature*, **346**, 470–471.

LUONG, T.T. (1986). Vietnam: Ho Chi Minh City, 1976–1981. In *Cancer Occurrence in Developing Countries* (IARC Scientific Publications No. 75), Parkin, D.M. (ed.) pp. 309–311. International Agency for Research on Cancer: Lyon.

LUONG, T.T. & PHAM, T.L. (1964). Le cancer au nord Viet-Nam (de 1955 à 1961). *Acta Un. Int. Cancer*, **20**, 623–625.

MUÑOZ, N. & BOSCH, F.X. (1992). HPV and cervical neoplasia: review of case-control and cohort studies. In *The Epidemiology of Human Papillomavirus and Cervical Cancer* (IARC Scientific Publications No. 119), Muñoz, N., Bosch, F.X., Shah, K.V. & Meheus, A. (eds) pp. 251–261. International Agency for Research on Cancer: Lyon.

OLIVIER, S. & PARKIN, D.M. (1992). *CANREG: Cancer Registration Software for Microcomputers* (IARC Internal Report No. 92/004). International Agency for Research on Cancer: Lyon.

PARKIN, D.M. & MUIR, C.S. (1992). Comparability and quality of data. In *Cancer Incidence in Five Continents, Volume VI* (IARC Scientific Publications No. 120), Parkin, D.M., Muir, C.S., Whelan, S.L., Gao, Y.T., Ferlay, J. & Powell, J. (eds) pp. 45–173. International Agency for Research on Cancer: Lyon.

PARKIN, D.M., MUIR, C.S., WHELAN, S.L., GAO, Y.T., FERLAY, J. & POWELL, J. (eds) (1992). *Cancer Incidence in Five Continents, Volume VI* (IARC Scientific Publications No. 120). International Agency for Research on Cancer: Lyon.

PARKIN, D.M., PISANI, P. & FERLAY, J. (1993). Estimates of the worldwide incidence of eighteen major cancers in 1985. *Int. J. Cancer*, **54**, 594–606.

ROSS, R.K., BERNSTEIN, L., HARTNETT, N.M. & BOONE, J.R. (1991). Cancer patterns among Vietnamese immigrants in Los Angeles County. *Br. J. Cancer*, **64**, 185–186.

SMITH, P.G. (1992). Comparison between registries: age-standardized rates. In *Cancer Incidence in Five Continents, Volume VI* (IARC Scientific Publications No. 120), Parkin, D.M., Muir, C.S., Whelan, S.L., Gao, Y.T., Ferlay, J. & Powell, J. (eds) pp. 865–870. International Agency for Research on Cancer: Lyon.

SRIAMPORN, S., VATANASAPT, V., PISANI, P., YONGCHAIYUDHA, S. & RUNGPITARANGSRI, V. (1992). Environmental risk factors for nasopharyngeal carcinoma: a case-control study in northeastern Thailand. *Cancer Epidemiol. Biomark. Prev.*, **1**, 345–348.

SWERDLOW, A.J. (1991). Mortality and cancer incidence in Vietnamese refugees in England and Wales: a follow-up study. *Int. J. Epidemiol.*, **20**, 13–19.

TOMATIS, L., AITIO, A., DAY, N.E., HESELTINE, E., KALDOR, J., MILLER, A.B., PARKIN, D.M. & RIBOLI, E. (eds) (1990). *Cancer: Causes, Occurrence and Control* (IARC Scientific Publications No. 100). International Agency for Research on Cancer: Lyon.

TUNG, T.T. (1973). Le cancer primaire du foie au Viet-Nam. *Chirurgie*, **99**, 427–436.

UNDP (1992). *Human Development Report 1992*. Oxford University Press: New York.

VAN, D.D. (1984). Herbicides as a possible cause to liver cancer. In *Herbicides in War: The Long-Term Ecological and Human Consequences*, Westing, A.H. (ed.) pp. 119–121. Taylor & Francis: London.

VATANASAPT, V., TANGVORAPHONKCHAI, V., TITAPANT, V., PIPITGOOL, V., VIRIYAPAP, D. & SRIAMPORN, S. (1990). A high incidence of liver cancer in Khon Kaen province, Thailand. *S.E. Asian J. Trop. Med. Publ. Hlth*, **21**, 489–494.

WHELAN, S.L., PARKIN, D.M. & MASUYER, E. (eds) (1990). *Patterns of Cancer in Five Continents* (IARC Scientific Publications No. 102). International Agency for Research on Cancer: Lyon.

YU, M.C., HO, J.H.C., LAI, S.H. & HENDERSON, B.E. (1986). Cantonese-style salted fish as a cause of nasopharyngeal carcinoma: report of a case-control study in Hong Kong. *Cancer Res.*, **46**, 956–961.

YU, M.C., MO, C.C., CHONG, W.X., YEH, F.S. & HENDERSON, B.E. (1988). Preserved foods and nasopharyngeal carcinoma: a case-control study in Guangxi, China. *Cancer Res.*, **48**, 1954–1959.